# Perspectives of Healthcare Professionals on the Management of Patients With Tracheostomy

**DOI:** 10.7759/cureus.82051

**Published:** 2025-04-10

**Authors:** Alexandros Ganaiem, Alexandra Skitsou, George Charalampous

**Affiliations:** 1 Master of Public Health, Healthcare Administration and Management, Frederick University, Nicosia, CYP; 2 Otolaryngology - Head and Neck Surgery Department, Sotiria Thoracic Diseases Hospital of Athens, Athens, GRC; 3 Emergency Department, Hippocratio General Hospital, Athens, GRC

**Keywords:** healthcare professionals, healthcare system, multidisciplinary tracheostomy team, otorhinolaryngology, patients management, quality of care provided, tracheostoma care, tracheostomy, tracheostomy complications, tracheostomy placement

## Abstract

Introduction

Tracheostomy is a critical and common intervention for respiratory support. The complexity of cases requires vigilance, skilled personnel, and close interdisciplinary collaboration.

Objective

This study aims to highlight the complexity and interdisciplinary nature of tracheostomy care. By exploring healthcare professionals' perspectives on which specialties are most suitable for each stage of management, the study seeks to identify current clinical practices, uncover limitations, and suggest improvements in care delivery.

Methodology

The first part of the study involved a literature review, providing a scientific overview of the essential and multifaceted demands of tracheostomy care. In the second part, a quantitative approach was applied, utilizing a well-structured questionnaire to collect data from 101 healthcare professionals regarding their views on tracheostomy management. The research was conducted from September 1, 2023, to October 20, 2023, in public hospitals in Athens, Greece. The questionnaires were electronically distributed via Google Forms, and responses were analyzed using IBM SPSS Statistics for Windows, Version 27 (Released 2020; IBM Corp., Armonk, NY, USA).

Results

The literature indicates that tracheostomy cases are encountered frequently in clinical practice and require complex, specialized, and multidisciplinary care. Despite their frequency and impact, there is a lack of standardized guidelines for their management, leading to inconsistent practices and preventable complications, often resulting in prolonged hospitalizations, Intensive Care Unit (ICU) readmissions, increased healthcare costs, and elevated morbidity and mortality rates. The questionnaire examined 20 key clinical actions across different care stages. Most participants identified the otolaryngologist as the most appropriate for half of these tasks, including tracheostomy placement, patient education, tube selection, cuff decisions, fistula management, hygiene, swallowing assessment, and cuff explanation. Nurses were associated with direct patient care - stoma management, suctioning, oral hygiene, and safe feeding practices. Speech therapists were linked to communication support and alternative communication training. Pulmonologists were assigned respiratory function oversight, dietitians, nutrition planning, and psychologists, emotional support. Research hypotheses were developed to explore differences in opinions based on demographic and professional variables, including gender, age, education, experience, specific training, and engagement with current practices. These findings emphasized both the importance of experience and the lack of standardized role definitions in tracheostomy care.

Conclusions

Tracheostomy management places a significant burden on healthcare systems due to its intensive and multifaceted demands. Effective resource and personnel management can reduce complications, enhance patient safety and the quality of care, and improve patient outcomes, while simultaneously contributing to the economic sustainability of the healthcare system. While the specialization of Otorhinolaryngology appears most suitable for many actions, there is a recognized need for other professionals for specific vital tasks. It is the responsibility of healthcare units to establish interdisciplinary teams with specialized knowledge, proper training, and clearly defined roles to ensure safe, efficient, and high-quality care delivery.

## Introduction

Respiratory failure (RF) and its management with invasive mechanical ventilation (IMV) are among the most common diagnoses in adult patients admitted to the Intensive Care Unit (ICU), with over 90% of these patients requiring full ICU support [1]. In patients with RF who require prolonged IMV with an endotracheal tube, a tracheostomy may be performed to reduce patient discomfort and prevent potential complications associated with prolonged endotracheal tube use. These complications may include ventilator-associated pneumonia, extended sedation, pressure ulcers, direct anatomical damage, delirium, and muscle weakness [2].

The word "tracheostomy" originates from the Greek words "trachea arteria" (meaning rough artery) and "tome" (meaning cut). Tracheostomy is, therefore, an opening (incision) made on the anterior surface of the neck and the trachea, through which a tube (tracheostomy tube) is inserted and secured to the neck. The distal end of the tube resides within the trachea and serves as an alternative airway for patients who require it.

Tracheostomy is one of the most common procedures performed in ICU patients with prolonged RF and IMV [3]. A study by Mehta et al. showed that age-adjusted tracheostomy rates among all mechanical ventilation patients increased from 16.7 to 34.3 cases per 100,000 adults between 1993 and 2012 [4]. More than 12,000 tracheostomies are performed annually in the United Kingdom, and approximately 20% of ICU patients are treated with a tracheostomy [5]. McGrath et al. further report that tracheostomies serve as artificial airways for approximately 15,000 patients annually in England and Wales [6]. These procedures include both surgical and percutaneous insertions, whether planned or emergency operations. Consequently, these complex and demanding patients are increasingly encountered in ICUs and surgical settings [7].

Despite its benefits, tracheostomy is associated with significant morbidity and mortality, emphasizing the importance of skilled staff and structured care protocols. Tracheostomy patients are often critically ill and have an in-hospital mortality rate ranging from 25% to 60%, with most of this mortality attributed to underlying disease. However, up to 30% of tracheostomy patients experience an adverse event during hospitalization, and measurable complications occur in 60%-70% of such incidents, including hospital admissions or ICU readmissions, prolonged hospital stays, hypoxic brain injury, and death [6]. Delays in care are common due to the variety and complexity of services accessed by tracheostomy patients [6]. Key studies underscore failures in the care of hospitalized tracheostomy patients, highlighting how inadequate staff training, insufficient equipment, and a lack of essential infrastructure contribute to preventable harm, increased morbidity and mortality, as well as a significant financial burden on healthcare systems.

The article provides a brief review of the definition, indications, contraindications, and complications of tracheostomy, along with tube characteristics, care techniques, and the importance of multidisciplinary teams. Additionally, it presents an analysis of a prospective observational study conducted in public hospitals in Athens, Greece, in 2023, focusing on the perspectives of healthcare professionals on tracheostomy management. The research examines their views on identifying the most appropriate practitioners for each stage of care and the management of complex tracheostomy patients.

This study aims to highlight the complexity and interdisciplinary nature of tracheostomy care. By exploring which healthcare professionals are best suited for each stage of management, the study intends to identify current clinical practices, expose limitations, and suggest improvements in care delivery. When each intervention and procedure is performed by the most appropriate specialist, the result is a comprehensive and high-quality approach that integrates the expertise of multiple disciplines. Strengthening collaboration among specialties, establishing clarity in roles and responsibilities, and improving coordination are essential for optimizing patient outcomes. Such an approach can significantly reduce the incidence of complications, minimize unnecessary healthcare expenditures - such as prolonged hospital stays, ICU readmissions, and excessive medication and supply use - and ultimately lower morbidity and mortality rates. This approach seeks to tackle challenges in adopting an updated guide to ensure reliable improvements in patient care and outcomes.

## Materials and methods

The first part of the study involved a literature review to scientifically explain the importance and complexity of these cases and investigate the current stance of healthcare units. For this literature review, Greek and international sources were consulted. The search strategy included several databases: for international literature, sources such as PubMed, EBSCO, the World Health Organization (WHO) website, Google Scholar, and ResearchGate were utilized. Greek literature was accessed through platforms like Google Scholar and the National Documentation Centre. Additionally, relevant nursing and medical textbooks focusing on tracheal anatomy and tracheostomy management were reviewed.

The following search terms were used for the Greek literature: "τραχειοστομία" (tracheostomy), "τραχειοτομή" (tracheotomy), "τραχειοσωλήνας" (tracheal tube), "ασθενείς ΜΕΘ" (ICU patients), and "επαγγελματίες υγείας" (healthcare professionals). Corresponding English search terms included: "tracheostomy," "tracheotomy," "tracheal tube," "ICU patients," and "healthcare professionals."

The primary inclusion criterion for scientific articles was publication within the last 10 years. However, older academic texts were also included when relevant to the anatomical or foundational aspects of the subject. A total of 289 relevant scientific articles were retrieved using various combinations of Boolean operators. Titles were initially screened to assess relevance. The main criterion for inclusion was the alignment of the article's content with the topic of tracheostomy management. Unofficial publications, clinical guides, and sources not meeting established scientific standards were excluded to ensure the validity and rigor of the literature review.

In the second part, a quantitative approach was chosen, utilizing a well-structured questionnaire to collect data on the opinions of healthcare professionals regarding the management of patients with tracheostomy. The questionnaire included a total of 31 questions: three open-ended questions and 28 closed-ended questions, one of which had a sub-question. The questions were organized around two main areas: (a) 11 questions about demographic characteristics, and (b) 20 questions about the personal perceptions of respondents regarding the identification of the most suitable specialist for 20 different stages of treatment and care for patients with tracheostomy (see Appendices, Table 2).

The population consisted of 101 healthcare professionals involved in various stages of care for tracheostomy patients, working in public hospitals located in Athens, Greece. The research was conducted from September 1, 2023, to October 20, 2023. Questionnaires were distributed electronically using Google Forms, and the data were subsequently analyzed using IBM SPSS Statistics for Windows, Version 27 (Released 2020; IBM Corp., Armonk, NY, USA).

The dissemination of the questionnaire was carried out using the snowball sampling method, where initial participants were asked to forward the questionnaire to their colleagues within the healthcare system. This method, which relies on peer-to-peer distribution, is especially effective in reaching hard-to-access populations and expanding the sample across different specialties and institutions. It allowed the study to include participants from a range of public hospitals in Athens, capturing a more holistic and representative view of healthcare professionals, rather than being limited to a single hospital’s approach. The choice to focus on public hospitals was deliberate, as these facilities frequently manage patients with complex needs and comorbidities. Such cases often require multidisciplinary care and the availability of specialized units, such as ICUs, emergency departments, and surgical departments - resources more commonly found in public sector institutions in Greece. These hospitals also encounter a higher volume of acute and critical cases, providing a relevant context for the study’s objectives.

The study sought to include a wide range of healthcare professionals involved in the day-to-day management of such patients. Participants included physicians (e.g., ENT specialists), nurses, occupational therapists, and physiotherapists. To ensure the validity and reliability of the collected data, only those verified as healthcare professionals, based on responses to demographic questions at the beginning of the survey, were included. Responses from individuals who did not meet the inclusion criteria were excluded from the analysis.

As detailed earlier, the complexity of tracheostomy patients highlights the need for multifaceted care. The purpose of the research is to explore healthcare professionals’ views on identifying the most suitable specialist for the various stages of treatment and care for patients with tracheostomy. Specifically, the study examines healthcare professionals' opinions on who is best suited to handle 20 specific actions (see Appendices, Table 2). The study aims to cover the most important stages of treatment, care, and management, as well as potential barriers and complications.

Recognizing the critical role of healthcare professionals’ expertise and experience, the research also investigates whether their perspectives differ based on demographic factors such as gender, age, education level, professional experience, previous training on tracheostomy management, and awareness of current developments.

Statistical hypotheses were formulated based on the sample data, focusing on the distributional behavior of the variables under study. The demographic data were categorized, and statistical hypotheses were then used to determine whether statistically significant differences existed between the categories regarding the survey questions. The null hypothesis (H0) posited no association between the categorical variable and the response regarding the tracheostomy care action under study, while the alternative hypothesis (H1) - when H0 is rejected - proposed the existence of a statistically significant relationship. For each hypothesis, the Pearson Chi-square test (χ²) was conducted using IBM SPSS Statistics for Windows, Version 27, to assess potential associations, thereby guiding inferential decision-making and the derivation of final conclusions. Prior to conducting the tests, data preprocessing procedures, such as cell merging and variable renaming, were undertaken to ensure a complete representation of all response categories. Then, the Chi-square test of independence for two categorical variables was applied in the subsequent hypothesis evaluations.

This study seeks to determine the general consensus among healthcare professionals on the responsibilities of each specialist, evaluating the clarity of roles and collaboration in supporting tracheostomy patients. Discrepancies in knowledge and opinions could indicate gaps in training, role allocation, and interdisciplinary teamwork, potentially impacting patient care quality, safety, and the efficiency of healthcare units.

Ethical considerations and limitations

The questionnaire used in this research was provided with the written consent of its authors. It was originally developed in Greek in 2015 by Chrysa Kateroudaki, Spyridoula Mourkogianni, and Mary Nikolaou as part of their undergraduate thesis, "The Views of Healthcare Professionals on Tracheostomy Management," at the Department of Speech Therapy, Faculty of Health and Welfare Professions, Technological Institute of Western Greece in Patras (see Appendices, Table 3) [8]. Their research focused on the perspectives of healthcare professionals regarding tracheostomy management. The questionnaire was granted exclusively for this study, with permission for use and distribution to participants, ensuring their anonymity.

The questionnaire used in this study was adapted into Google Forms for ease of distribution and translated into English for the purposes of the study, and potential future use, contingent upon appropriate consent.

A pilot study was not conducted prior to data collection, as the questionnaire had already been applied in previous research contexts. Its clarity and relevance were considered sufficient. Nonetheless, expert review and iterative feedback were used to ensure clarity and face validity in the adapted version.

Participation in the study was voluntary, and informed consent was obtained through an introductory statement presented at the beginning of the questionnaire. This text clearly outlined the aim of the study, explained the inclusion and exclusion criteria (specifically targeting healthcare professionals in public hospitals), and emphasized that the data would be used solely for this research. Anonymity was strictly maintained: no identifying personal data were collected, and responses were stored securely in encrypted digital files, accessible only to the primary researchers. All collected information was handled securely to prevent privacy breaches.

Questionnaires were carefully designed to avoid misleading or influencing participants, and efforts were made to include a diverse sample representing a wide range of healthcare professionals. The questions were appropriate and avoided sensitive or offensive topics.

This study did not involve human subjects as defined by IRB guidelines, as it was a survey of healthcare professionals without personal data collection. According to IRB guidelines, this study meets the criteria for exemption from IRB approval. The research was conducted using questionnaires sent to healthcare professionals, which solely contained questions regarding their opinions and professional practices. No personal or identifiable data of the participants were collected, nor were any patient-related data, medical records, biological samples, or treatment-related information included in the study. Furthermore, the study was not clinical or laboratory-based and did not involve human or animal subjects in a biomedical or interventional context. All responses were anonymous, ensuring the confidentiality of participants. Therefore, the study was conducted in accordance with IRB and journal guidelines, which indicated that, due to its non-interventional and anonymous nature, IRB approval was not required.

Key strengths of the study include the use of a methodology that safeguarded respondent anonymity, the inclusion of a diverse group of healthcare professionals, and the analysis of correlations between key variables and participants’ demographic and professional characteristics, reinforcing the study’s reliability.

As with any research, several limitations must be acknowledged. First, the sample size (n = 101) is sufficient for preliminary exploration, but future research with larger samples is needed to enhance the robustness and generalizability of the findings. Second, the geographic scope was limited to Athens, Greece. Although Athens is home to a significant concentration of the country’s public healthcare infrastructure and patient population, expanding the study to other regions and countries would provide more comprehensive insights. Moreover, a limitation of the study is the lack of direct questions addressing interdisciplinary collaboration.

Additionally, the electronic distribution method may have introduced bias, as only individuals familiar with digital tools like Google Forms may have participated. This could have excluded professionals with limited digital literacy or access. Moreover, due to the snowball sampling method, the exact response rate could not be calculated - a known limitation of this technique. While snowball sampling effectively broadens the sample and includes diverse perspectives, it lacks randomization and introduces potential selection bias, as participants tend to recruit individuals within their own professional circles.

Despite these limitations, the methodological transparency and acknowledgment of potential biases provide a solid foundation for future research in this area, offering insights for study design improvements and broader applicability.

## Results

Baseline characteristics

**Figure 1:**
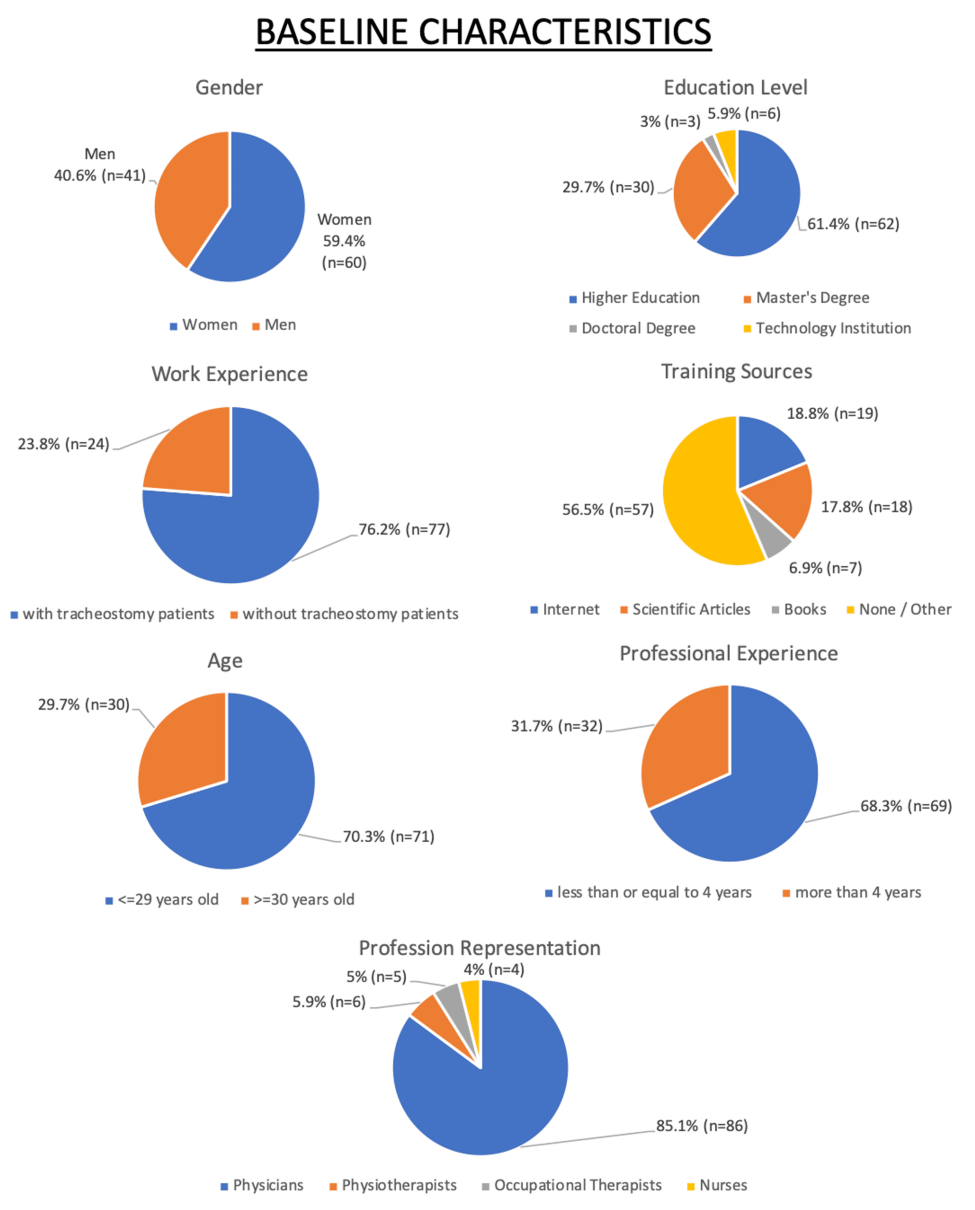
Baseline Characteristics

The study included a diverse sample of healthcare professionals, with 60 (59.4%) of participants being women and 41 (40.6%) men. The majority (71, or 70.3%) were under the age of 30, while 30 (29.7%) were above 30 years old. Regarding education level, 62 (61.4%) had completed higher education, 30 (29.7%) held a master’s degree, and three (3.0%) had a doctoral degree. A small percentage (6, or 5.9%) had obtained qualifications from a Technological Educational Institute (TEI) (Figure 1).

Most participants had limited professional experience, with 69 (68.3%) reporting four years or less in their field. When asked about their sources of training and information on tracheostomy care, 19 (18.8%) respondents cited the internet, 18 (17.8%) scientific articles, and seven (6.9%) books as their primary sources (Figure 1).

Notably, 77 (76.2%) of participants had prior experience working with tracheostomy patients. The sample consisted mainly of physicians from various specialties (Figure 1).

Healthcare professionals’ perspectives on responsibility at each stage

**Figure 2:**
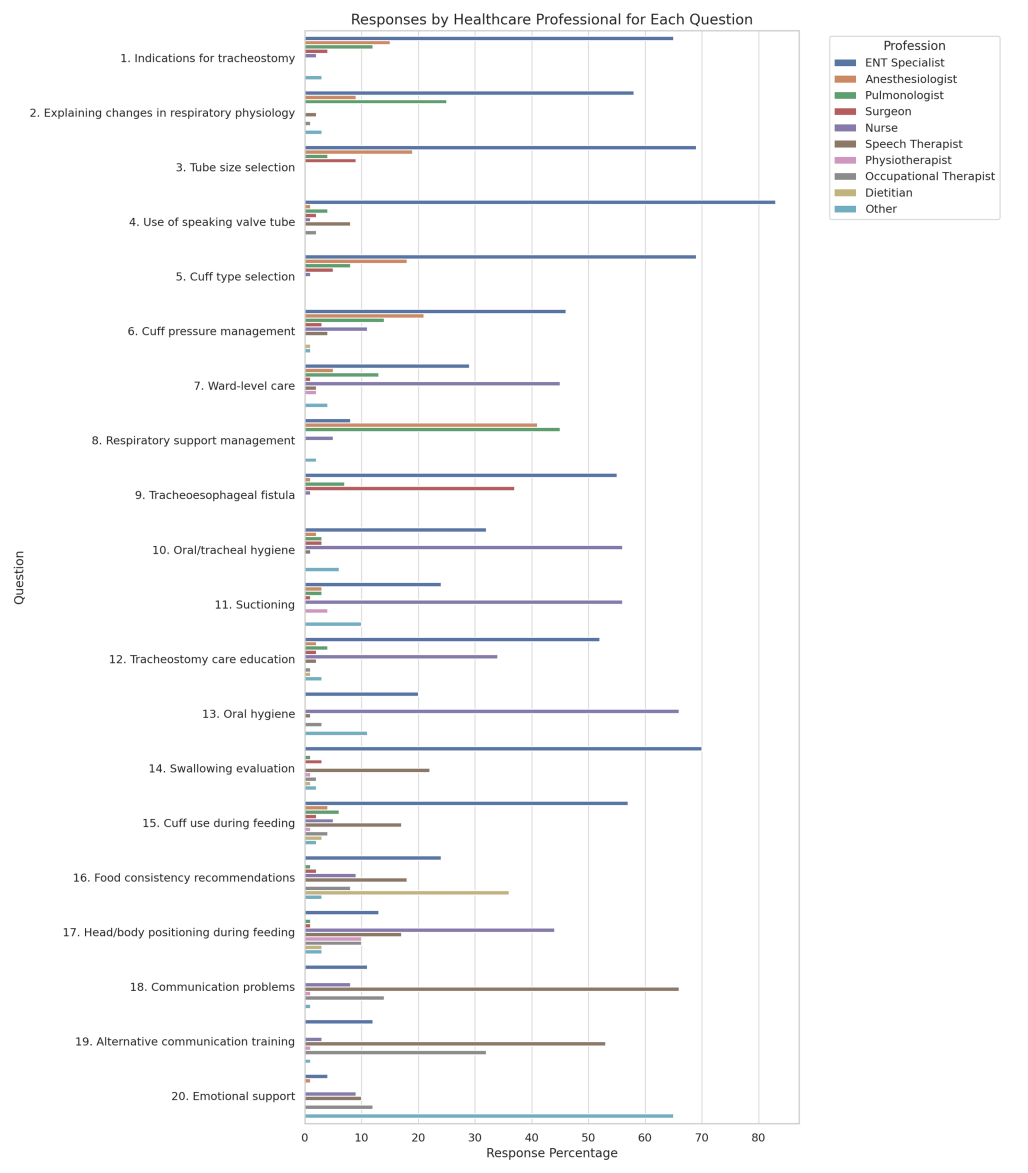
Healthcare Professionals’ Perspectives on Responsibility at Each Stage

Participants provided insights into the roles of different healthcare professionals in tracheostomy care, highlighting which specialists were considered most suitable for specific tasks (Figure 2).

Role of the Otorhinolaryngologist (ENT Specialist)

The ENT specialist was identified as the most appropriate healthcare professional for half of the evaluated tasks. This included determining the indications for tracheostomy placement, informing patients about physiological changes in respiratory function post-procedure, selecting the internal/external diameter and length of the tracheostomy tube, and deciding on the use of fenestrated tubes for speech production. ENT specialists were also considered key figures in determining the type of cuff to be used, adjusting cuff pressure, and managing complications such as tracheoesophageal fistulas. Additionally, decisions regarding educating and instructing patients on hygiene and care protocols for tracheostomy, assessing the patient’s swallowing ability, and explaining the use of the cuff during feeding were predominantly assigned to this specialty.

Role of the Nurse

Nurses were primarily associated with bedside patient care, including tracheostomy stoma management, suctioning, and maintaining oral hygiene. They were also considered responsible for ensuring proper patient positioning during and after feeding, which plays a crucial role in minimizing aspiration risks.

Role of the Speech Therapist

The role of the speech therapist was mainly focused on communication support. They were identified as the most suitable professionals for assisting patients with communication difficulties, and training them to use alternative communication methods until normal function was restored.

Role of Other Specialties

Pulmonologists were primarily responsible for managing respiratory function through mechanical support, while psychologists were considered the most suitable professionals for addressing the emotional challenges faced by tracheostomy patients.

Although no dietitians participated in this study, it is important to clarify that dietitians are indeed employed in most public hospitals in Athens and throughout Greece. Their absence in the sample is likely due to the limitations of the snowball sampling method and is not indicative of their absence in clinical practice. Despite this, other healthcare professionals - drawing from their clinical experience, academic training, ongoing education, and daily collaboration with dietitians - are well-informed about the role dietitians play in multidisciplinary care. Their responses reflect an informed perspective on the appropriateness of dietitians in managing aspects such as recommending safe food consistencies, particularly in tracheostomy patients. Therefore, the findings regarding the perceived suitability of dietitians remain valid, even in the absence of direct responses from this professional group. Future studies may benefit from targeted recruitment strategies to ensure the inclusion of underrepresented specialties like dietetics.

Statistical analysis

To explore potential differences in perspectives, statistical analyses were conducted based on variables such as gender, age, education level, professional experience, prior training in tracheostomy care, and engagement with updates in the field. Statistical hypotheses were formulated based on the sample data, focusing on the distributional behavior of the variables under study. Several statistically significant differences emerged in professionals’ opinions regarding specific responsibilities.

Statistical Analysis of Factors Influencing Professional Opinions

Experience with tracheostomy patients was found to influence perceptions of cuff type selection, with those who had prior experience being more likely to consider ENT specialists as the most suitable professionals for this task (p < 0.01). Similarly, physicians (p < 0.05), professionals with postgraduate education (p < 0.05), those aged 28 and above (p < 0.01), and those with five or more years of work experience (p < 0.05) were more inclined to assign the responsibility of cuff pressure adjustment to ENT specialists. Participants who actively followed tracheostomy advancements were more likely to consider ENT specialists as the appropriate professionals for tasks such as identifying tracheostomy indications (p < 0.05). Additionally, those who followed tracheostomy advancements (p < 0.05) or held higher academic qualifications (p < 0.05) were more likely to consider ENT specialists as the most suitable professionals for tube diameter and length selection. Speech production with fenestrated tubes was also primarily attributed to ENT specialists, especially by physicians (p < 0.01).

Differences also emerged in the perspectives on tracheostomy stoma care and suctioning. Physicians tended to view ENT specialists as the most suitable for stoma care (p < 0.05), while those with postgraduate education considered nurses the primary professionals responsible for suctioning (p < 0.05). Regarding hygiene education, professionals with prior experience in tracheostomy care were more likely to assign this role to nurses (p < 0.01). Moreover, participants who had not received specific training in tracheostomy care (p < 0.05) were more likely to consider nurses the most appropriate healthcare professionals to be responsible for proper body and head positioning during and after feeding, compared to professionals who had received specialized training. On the other hand, professionals with a master’s or doctoral degree were significantly less inclined (p < 0.001) to view nurses as the most suitable professionals for managing patient positioning during feeding, compared to those with a higher education/technological institution background, where no such statistically significant association was found. Additionally, physicians are more likely to consider ENT specialists as the most suitable professionals to assess swallowing (p < 0.001), and to inform patients about physiological changes in respiratory function post-procedure (p < 0.05). In terms of emotional support, non-physicians were more likely to assign this role to psychologists (p < 0.001), highlighting the recognition of specialized mental health care in tracheostomy patient management.

Finally, professionals with postgraduate education (p < 0.05) were less likely to consider dietitians as the most appropriate professionals for recommending safe food consistency. Similarly, those with five or more years of experience (p < 0.05) were less likely to view dietitians as the most suitable for this role, compared to those with less than five years of experience.

No significant differences were observed based on gender, indicating that professional perspectives on tracheostomy care were not influenced by this demographic factor (Table 1).

**Table 1:** Statistical Analysis of Factors Influencing Professional Opinions Statistical Hypothesis: The null hypothesis (H0) posited no association between the categorical variable and the response regarding the tracheostomy care action under study, while the alternative hypothesis (H1) - when H0 is rejected - proposed the existence of a statistically significant relationship.

Categorical Variables	Category A	Population A% (n)	Category B	Population B% (n)	Tracheostomy Care Actions Under Study	Response A% (n)	Response B% (n)	Pearson Chi-Square (χ²)	p-value	Statistical Hypothesis
Education Level	Master's or Doctoral Degree	32.7% (n = 33)	Higher Education or Technology Institution	67.3% (n = 68)	Selection of tracheostomy tube diameter and length	84.8% (28/33) - Otolaryngologist	60.3% (41/68) - Otolaryngologist	6.189	0.013	H1
Education Level	Master's or Doctoral Degree	32.7% (n = 33)	Higher Education or Technology Institution	67.3% (n = 68)	Cuff pressure measurement (inflation/deflation)	60.6% (20/33) - Otolaryngologist	38.2% (26/68) - Otolaryngologist	4.483	0.034	H1
Education Level	Master's or Doctoral Degree	32.7% (n = 33)	Higher Education or Technology Institution	67.3% (n = 68)	Performing suctioning procedure	69.7% (23/33) - Nurse	48.5% (33/68) - Nurse	4.03	0.045	H1
Education Level	Master's or Doctoral Degree	32.7% (n = 33)	Higher Education or Technology Institution	67.3% (n = 68)	Recommendation of food consistency for safe oral intake	78.8% (26/33) - No Dietitian	57.4% (39/68) - No Dietitian	4.45	0.035	H1
Education Level	Master's or Doctoral Degree	32.7% (n = 33)	Higher Education or Technology Institution	67.3% (n = 68)	Responsibility for proper body and head positioning during and after feeding	81.8% (27/33) - No Nurse	44.1% (30/68) - No Nurse	12.844	0.00034	H1
Specific Training in Tracheostomy	No specific training	82.2% (n = 83)	Specific training	17.8% (n = 18)	Responsibility for proper body and head positioning during and after feeding	49.3% (41/83) - Nurse	16.7% (3/18) - Nurse	6.446	0.011	H1
Keeping Up With Tracheostomy Developments	Keeping Up	43.6% (N = 44)	Don't Keep Up	56.4% (n = 57)	Identifying indications for tracheostomy placement	77.3% (34/44) - Otolaryngologist	54.4% (31/57) - Otolaryngologist	5.67	0.017	H1
Keeping Up With Tracheostomy Developments	Keeping Up	43.6% (N = 44)	Don't Keep Up	56.4% (n = 57)	Selection of tracheostomy tube diameter and length	81.8% (36/44) - Otolaryngologist	57.9% (33/57) - Otolaryngologist	6.566	0.01	H1
Physicians or Other Healthcare Professionals	Physicians	85.1% (n = 86)	Other Healthcare Professionals	14.9% (n = 15)	Informing patients about respiratory function changes post-procedure	61.6% (53/86) - Otolaryngologist	33.3% (5/15) - Otolaryngologist	4.182	0.041	H1
Physicians or Other Healthcare Professionals	Physicians	85.1% (n = 86)	Other Healthcare Professionals	14.9% (n = 15)	Use of fenestrated tubes for speech production	87.2% (75/86) - Otolaryngologist	53.3% (8/15) - Otolaryngologist	10.008	0.002	H1
Physicians or Other Healthcare Professionals	Physicians	85.1% (n = 86)	Other Healthcare Professionals	14.9% (n = 15)	Cuff pressure measurement (inflation/deflation)	50% (43/86) - Otolaryngologist	20% (3/15) - Otolaryngologist	4.635	0.031	H1
Physicians or Other Healthcare Professionals	Physicians	85.1% (n = 86)	Other Healthcare Professionals	14.9% (n = 15)	Tracheostomy stoma care	36% (31/86) - Otolaryngologist	6.7% (1/15) - Otolaryngologist	5.09	0.02	H1
Physicians or Other Healthcare Professionals	Physicians	85.1% (n = 86)	Other Healthcare Professionals	14.9% (n = 15)	Swallowing assessment	76.7% (66/86) - Otolaryngologist	26.7% (4/15) - Otolaryngologist	15.057	0.0001	H1
Physicians or Other Healthcare Professionals	Physicians	85.1% (n = 86)	Other Healthcare Professionals	14.9% (n = 15)	Providing emotional care to manage psychological challenges	43% (37/86) - Psychologist	80% (12/15) - Psychologist	6.991	0.008	H1
Age Influence	≽28 years old	48.5% (n = 49)	≼27 years old	51.5% (n = 52)	Cuff pressure measurement (inflation/deflation)	59.2% (29/49) - Otolaryngologist	32.7% (17/52) - Otolaryngologist	7.139	0.008	H1
Professional Experience	≼4 years	68.3% (n = 69)	≽5 years	31.7% (n = 32)	Recommendation of food consistency for safe oral intake	42% (29/69) - Dietitian	21.9% (7/32) - Dietitian	3.871	0.049	H1
Professional Experience	≼4 years	68.3% (n = 69)	≽5 years	31.7% (n = 32)	Cuff pressure measurement (inflation/deflation)	37.7% (26/69) - Otolaryngologist	62.5% (20/32) - Otolaryngologist	5.430	0.02	H1
Work Experience With Tracheostomy Patients	YES	76.2% (n = 77)	NO	23.8% (n = 24)	Cuff type selection	75.3% (58/77) - Otolaryngologist	45.8% (11/24) - Otolaryngologist	7.352	0.007	H1
Work Experience With Tracheostomy Patients	YES	76.2% (n = 77)	NO	23.8% (n = 24)	Providing education on tracheostomy hygiene and care protocols	40.3% (31/77) - Nurse	12.5% (3/24) - Nurse	6.31	0.0012	H1
Work Experience With Tracheostomy Patients	YES	76.2% (n = 77)	NO	23.8% (n = 24)	Cuff pressure measurement (inflation/deflation)	51.9% (40/77) - Otolaryngologist	25% (6/24) - Otolaryngologist	5.357	0.021	H1

Summary of findings

The variation in responses reflects the diverse perspectives of healthcare professionals and highlights the division of responsibilities across specialties to ensure comprehensive care for tracheostomy patients. While the specialization of Otorhinolaryngology appears most suitable for many actions, there is a recognized need for other professionals for specific, vital tasks.

Furthermore, the results of the statistical analysis emphasize the importance of specialized training and experience in shaping professional opinions on tracheostomy care. The assignment of responsibilities varied depending on professional background, education level, and prior exposure to tracheostomy patients. These results suggest that role definitions within the facilities may not be clear. These findings support the need for clearly defined roles and standardized protocols by relevant experts within multidisciplinary tracheostomy teams (MTTs) to ensure comprehensive and effective patient care.

Tracheostomy is often perceived as a straightforward topic of discussion, given that it is a relatively safe, simple, and frequently life-saving procedure. However, if performed without careful foresight, proper planning, and thorough evaluation of the patient’s clinical condition, underlying pathology, and anatomy, or without adequate support and in a suboptimal environment, a tracheostomy can become a highly dangerous and potentially life-threatening procedure [9]. Furthermore, patients with tracheostomies place considerable demands on healthcare resources and personnel. Their care is complex and often requires long-term or even lifelong management. This complexity is further intensified by the risk of complications, particularly when care is inadequate or inconsistently delivered. These findings highlight the necessity of a well-coordinated, multidisciplinary approach to ensure optimal outcomes and minimize the burden on both the healthcare system and the patients themselves [9,10].

## Discussion

Indications and contraindications for tracheostomy

The most common indication for tracheostomy is to facilitate weaning from respiratory support in patients with acute RF and prolonged mechanical ventilation. Additional indications include pulmonary decongestion and suctioning for patients unable to clear excessive secretions (e.g., bulbar palsy or weak cough), airway protection for patients unable to maintain an open airway (e.g., various specific neurological conditions or reduced consciousness), and as part of a surgical procedure (e.g., laryngectomy). Other tracheostomy indications may be urgent, such as upper airway obstruction due to facial or laryngeal trauma, burns, anaphylaxis, infections, foreign bodies, or tumors.

However, there are certain contraindications for performing a tracheostomy, such as a platelet count <50,000 or coagulation disorders (platelet transfusion is recommended prior to the procedure: 1 unit per 10 kg of patient body weight until a platelet count above 50,000 is achieved), active bleeding in the cervical region, cervical trauma, thyroid goiter, cervical instability, abnormal anatomy, or tumors (contraindication to the percutaneous technique) [10-12].

Complications of tracheostomy

It is estimated that up to 30% of patients globally may experience a significant complication associated with tracheostomy. The complications of tracheostomy surgery are classified into early complications, which are related to the surgical procedure, and late complications, which are associated with patient care, the tracheostomy stoma, and the tracheostomy tube [13].

Complications related to the tracheostomy procedure include pneumothorax (1%-5%), vascular injury with major bleeding (up to 5%), injury to the thyroid isthmus (<1%), recurrent laryngeal nerve injury (<1%), misplacement of the tracheostomy tube (<1%), subcutaneous emphysema (<1%), esophageal injury leading to tracheoesophageal fistula (<1%), tracheal rupture (<1%), tracheal stenosis (<1%), and hypoxia and cardiopulmonary arrest (<1%) [10,12,14].

Complications related to tracheostomy care include tracheostomy tube obstruction, surgical site infection, tracheobronchitis, tracheomalacia, granuloma formation, esophageal necrosis, tracheoesophageal fistula, and tracheal stenosis [11,15,16].

Tracheostomy tube selection and management

The care of patients depends on multiple factors and is complex, requiring not only the involvement of various specialties but also the appropriate materials. For instance, the selection of the tube to be used is influenced by several considerations. Tracheostomy tubes come in a wide range of materials, sizes, and designs. Healthcare professionals must understand these differences to select the most suitable tube for each patient’s needs, while ensuring patient safety.

Tubes are made of either metal (silver or stainless steel) or plastic (polyvinyl chloride (PVC), silicone, or polyurethane). Metal tubes are durable, resistant to biofilm formation, easy to sterilize, and cost-effective for long-term use. However, they are rigid, lack a cuff, and may damage the trachea due to temperature changes, making them unsuitable for patients undergoing radiotherapy. PVC tubes adapt to body temperature and anatomy, while silicone tubes are soft, resistant to biofilm, and sterilizable. Tubes may also have a single or double lumen. Double-lumen tubes allow periodic cleaning without removing the main tube. The inner tube can be replaced, maintaining airway patency, though the reduced diameter increases breathing effort. Tubes can also be angled, standard, extra-length, or adjustable-length, depending on the patient’s anatomy or surgical needs.

The International Organization for Standardization (ISO) classifies tube size based on the internal diameter of the outer tube at its narrowest point. For adult females, tubes with an internal diameter of 6.0-6.5 mm are sufficient, while males typically require 7.0-8.0 mm. In children, the diameter of the fifth finger approximates tracheal size. Additionally, there are cuffed and cuffless tubes. Cuffed tubes create a sealed airway, using pressures of 20-30 cm H_2_O to prevent aspiration and maintain ventilation. Overinflation can damage the tracheal wall and impair swallowing reflexes. Cuffless tubes are used when mechanical ventilation is no longer required, provided the patient can breathe independently, swallow safely, and manage secretions effectively. Patients with cuffless or fenestrated tubes can use their fingers or a one-way valve to facilitate speech. Speech therapy may help new users learn to control airflow and produce speech efficiently. Proper maintenance of speaking tubes is essential for functionality and to prevent complications.

Key aspects of tracheostomy care

Hence, managing patients with a tracheostomy requires complex, multifaceted, and intensive care. These patients have diverse needs that can only be met through the involvement of skilled professionals, proper training, and effective collaboration. Below are some of the most critical aspects of tracheostomy care, aimed at optimizing patient outcomes and minimizing complications.

Suctioning

Errors during suctioning can lead to immediate or indirect complications, including hypoxia, distal granulation, ulcers, cardiovascular changes, pneumothorax, atelectasis, bacterial infections, and, in extreme cases, intracranial complications.

Care of the Tracheostomy Tube, Stoma, and Neck

Complications such as skin trauma, epidermolysis, vascular issues, and lymphatic malformations require meticulous attention.

Management of Tracheostomy Tube Cuff

Most initially inserted tracheostomy tubes have a cuff and are typically used short-term until the patient is weaned off mechanical ventilation and can manage secretions. Long-term use may be necessary for chronic conditions such as reduced consciousness or neuromuscular disorders affecting the pharynx. Excessively high cuff pressures increase the risk of tracheal stenosis, tracheomalacia, and tracheal fistulas, while low pressures can cause air leaks and allow micro-aspiration of secretions above the cuff.

Feeding and Swallowing

Therapy for dysphagia begins with a thorough evaluation of the patient’s condition, considering their medical history, underlying disease, structural observations, and the functionality of phonatory and articulatory organs. Treatment is initiated when the patient is hemodynamically stable. If feasible, oro-tracheal suctioning, sequential deflation of the cuff, and temporary occlusion of the tracheostomy tube are performed to redirect airflow through the upper airway, promoting the restoration of normal breathing and swallowing. For patients who cannot tolerate cuff deflation, it is essential to investigate the underlying reasons preventing this procedure.

Role of MTTs

Having all the above in mind, many researchers recommend that, in order to provide optimal care for these patients, the organization and management of an MTT are essential. Clair highlighted that inconsistencies in tracheostomy management pose a significant threat to patients' safety and to the healthcare system, resulting in hospital-acquired infections, prolonged hospital stays, and other complications, including mortality [17].

The lack of standardized care and associated complications have led many institutions to establish MTTs. These teams have been credited with significantly improving patient outcomes. Santos et al. reported numerous improvements in post-tracheostomy care and patient outcomes. These included shorter ICU stays, reduced overall hospital length of stay, accelerated ventilator weaning, fewer general tracheostomy-related complications, earlier initiation of feeding, and increased use of speaking valves [18].

Research, including studies by Holmes et al. and Sage Journals, emphasizes the critical role of various healthcare professionals in tracheostomy care. These include physiotherapists, speech and language therapists, occupational therapists, nurses, dietitians, psychologists, and physicians from diverse specialties, such as otolaryngology (ENT), pulmonology, intensive care, anesthesiology, and surgery [19]. Their collaboration is essential to address the multifaceted needs of tracheostomy patients comprehensively. The role of interdisciplinary care in tracheostomy management is critical and multifaceted, particularly in resource-constrained settings. Speech and language therapists contribute far beyond communication and swallowing support; their evaluation of secretion management and swallowing safety - often through instrumental assessments like FEES (Fiberoptic Endoscopic Evaluation of Swallowing) - can significantly influence decannulation decisions and reduce aspiration-related complications. Specialist nurses, originally focused on head and neck surgical patients, have expanded their expertise to non-surgical tracheostomy care, supporting general ward staff, reducing ICU readmissions, and improving overall outcomes. Their role overlaps with others yet enhances team collaboration and learning. The inclusion of dietitians is essential for assessing nutritional needs and determining safe feeding routes, often in coordination with physicians and speech therapists. Occupational therapists, though underrepresented in the literature, may aid in functional rehabilitation, particularly in patients with chronic conditions. Psychologists also play a vital role in addressing the emotional and cognitive consequences of ICU stays, communication loss, and altered body image, all of which may impact recovery and adherence. The integration of psychological support into tracheostomy care remains under-researched but appears beneficial. Moreover, successful transitional care programs involving interdisciplinary outreach teams (IDTs) have shown promise in reducing hospital readmissions by educating patients, caregivers, and community healthcare providers. Although medical professionals often retain overall responsibility for care coordination, non-hierarchical models of leadership within IDTs have been effective, promoting shared decision-making and enhancing care delivery. Expanding and clearly defining the roles of each team member - especially in under-resourced environments - can significantly improve outcomes, reduce complications, and support more sustainable healthcare practices [18,19]. 

The comprehensive analysis of the literature revealed that tracheostomy patient management is highly complex and demands a specialized, multidimensional, and resource-intensive approach. These cases require the coordinated involvement of multiple healthcare professionals across various disciplines to adequately address the diverse therapeutic and caregiving needs of the patients. In addition to human resources, tracheostomy care necessitates significant material support, including specialized equipment, appropriate infrastructure, and considerable time investment from healthcare personnel. The literature consistently associates these cases with high rates of morbidity and mortality. Moreover, complication rates are notably elevated, contributing to extended hospital stays, increased readmissions to hospital wards and ICUs, excessive medication use, and the potential need for further surgical interventions. These factors collectively impose a substantial burden on healthcare systems.

As a result, one of their key recommendations was the establishment of a dedicated MTT for the management of tracheostomy patients. This multidisciplinary approach ensures holistic care, improves patient safety, and enhances overall outcomes.

The aim of this study is to explore the perspectives of healthcare professionals regarding the management of tracheostomy patients. Understanding their views can help shape a structured allocation of tasks and a proper approach to caring for these patients, leveraging their education and experience.

As outlined in the study, the number of tracheostomy patients is steadily increasing due to various respiratory support needs. Daily, physicians of different specialties encounter tracheostomy patients, making it a concern for many healthcare and paramedical professionals. It is crucial to consider the perspectives of as many healthcare professionals as possible, across different specialties, to achieve a more holistic approach and draw comprehensive conclusions.

The questionnaire responses highlight the necessity of supporting patients through a multidisciplinary team with specialized knowledge, rather than relying solely on individual specialties. While ENT specialists were considered the most suitable for most tasks, the need for other professionals in vital roles was also emphasized. For instance, psychologists are crucial in providing emotional support, helping patients adapt to their new reality and changes in appearance. Speech therapists play a significant role in assisting communication, often collaborating with dietitians to oversee patients’ feeding. Nurses are indispensable for daily care, which helps reduce hospitalization time and the risk of complications, while pulmonologists contribute significantly to managing respiratory function.

These examples, along with the comprehensive insights of the study, underscore the importance of a multidisciplinary approach to addressing the varied needs of tracheostomy patients. Any intervention or procedure for a tracheostomy patient should be carried out by the most suitable professional, combining the expertise of multiple specialties to provide holistic and high-quality care. Vigilance, proper training, and standardized task allocation are essential to minimize inconsistencies in care and ensure equitable, high-quality management of these complex cases. Strengthening collaboration among different specialties and clarifying responsibilities are vital for reducing complications and alleviating the financial burden on the system, such as prolonged hospital stays, readmissions, excessive use of medications, materials, and personnel, ICU admissions, emergency surgeries, morbidity, and mortality.

Additionally, research hypotheses were raised regarding differences in the opinions of healthcare professionals based on gender, age, educational level, work experience, whether they have received any training on the subject, and whether they keep up with developments in tracheostomy to draw more specialized conclusions. This analysis highlights differences in healthcare professionals’ perspectives, emphasizing the importance of their experience and the lack of clarity in assigning complex responsibilities in tracheostomy management.

The conflicting responses among healthcare professionals highlight existing gaps in training, role allocation, and interdisciplinary teamwork - factors that may negatively impact the quality, safety, and overall efficiency of patient care. While there is often some degree of overlap in roles, allowing professionals from different specialties to partially compensate for each other, this substitution typically falls short of the care provided by the most appropriately trained and designated specialist. These disagreements suggest the absence of a clear and consistent protocol regarding which professional is responsible for each specific task in tracheostomy care. Such variation may depend on factors such as staff availability, equipment, or the experience level of each unit. Consequently, the effectiveness of patient management may be reduced. Studying the perspectives of healthcare professionals can help identify the most appropriate specialist for each stage of care. Furthermore, analyzing how scientific training and clinical experience relate to individual responses may guide the identification of optimal practices in areas where there is disagreement.

Drawing from their lifelong education and hands-on experience, healthcare professionals are well-positioned to contribute meaningfully to the development of effective tracheostomy care plans. Therefore, this study aims to evaluate the impact of existing practices, identify barriers, and propose strategies to enhance tracheostomy care. Furthermore, the study seeks to share adaptable methods for hospital and clinical practice while fostering discussions to develop an updated national guide for improving patient outcomes.

Current challenges and improvement measures in tracheostomy care

This study identified several pressing challenges in the management of tracheostomy patients in Athens' public hospitals, many of which align with international literature. A major issue is the lack of standardized protocols and clear task allocation among multidisciplinary teams. Variability in healthcare professionals' responses highlights inconsistent role distribution, often influenced by staffing, training, or institutional norms - contributing to fragmented care, increased complication rates, and reduced efficiency.

Gaps in specialized training - particularly in areas such as cuff management, suctioning, decannulation, oral hygiene, and safe feeding practices - pose additional safety risks. Perceptions of role suitability were significantly influenced by professional experience, education, and familiarity with tracheostomy advancements. For instance, those with prior hands-on experience were more likely to assign cuff selection and hygiene instruction to ENT specialists and nurses, respectively. These findings underscore the importance of experience-based training and suggest the need for structured mentorship and ongoing professional development to ensure knowledge transfer across all levels of clinical seniority.

Likewise, professionals with postgraduate education or those staying up-to-date with clinical developments assigned technical tasks - like tube selection and cuff pressure adjustment - to ENT specialists. These trends support the implementation of continuous education programs that emphasize evidence-based practices and promote familiarity with evolving guidelines.

Variations also emerged in basic, yet critical, care activities: suctioning was more often assigned to nurses by those with higher education, while physicians favored ENT specialists for stoma care. These findings highlight the need for clear task allocation within each institution, based on both competency and training background, to reduce subjective variability in the delivery of foundational daily care tasks.

Inadequate training was associated with inappropriate role assignments, such as patient positioning during feeding, underscoring the need for targeted educational interventions. This discrepancy suggests that targeted training in safe feeding techniques and aspiration prevention is needed across all specialties, to prevent the misallocation of responsibilities and potential complications. 

To improve care delivery, the study supports formal national or institutional guidelines, structured MTTs - including ENT specialists, nurses, speech therapists, dietitians, pulmonologists, and psychologists - and ongoing training across all specialties. Training modules should focus on critical care activities, including airway management, feeding safety, and communication strategies. Ongoing quality evaluation of care practices, using standardized assessment tools and quality indicators, may help reduce variability, lower complication rates, and improve healthcare system sustainability.

Furthermore, questionnaire responses may serve as a roadmap for building a standardized role allocation framework. By identifying the most frequently selected professionals for each task (Figure 2), the study provides data-informed insights into perceived role suitability. Given that these opinions stem from clinical experience and interdisciplinary collaboration, they can inform efforts to strengthen role clarity and coordination in tracheostomy care, offering a foundation for future comparisons and the development of a structured role allocation guide.

The findings of this study reveal certain differences in the perspectives of healthcare professionals regarding tracheostomy management, compared to previous research. Specifically, variations were observed when comparing the present results with those of Kateroudaki et al. [8], who examined healthcare professionals’ views on tracheostomy care. These discrepancies highlight differences in role assignments among various specialties in tracheostomy-related care and management.

One of the key differences pertains to hygiene and care protocols for tracheostomy management. While the present study indicates that 52 (51.5%) of respondents identified otolaryngologists (ENT specialists) as the primary professionals responsible for this aspect, the study by Kateroudaki et al. [8] suggested that nurses were more commonly recognized in this role. Similarly, regarding dietary recommendations for oral feeding, the present study found that 36 (35.6%) of respondents attributed this responsibility to dietitians, whereas the previous study identified speech and language therapists as the primary professionals involved.

Another notable divergence concerns the positioning and posture of the body and head during and after feeding. In this study, 44 (43.6%) of respondents indicated that nurses were responsible for ensuring proper positioning, whereas, in the findings of Kateroudaki et al. [8], occupational therapists were more commonly associated with this role. Differences also emerged in the use of alternative communication methods for tracheostomy patients, with 53 (52.5%) of respondents in the present study identifying speech and language therapists as the main professionals involved, while the previous study pointed to occupational therapists.

Furthermore, variations were observed in the provision of emotional support. The current study highlights psychologists as the primary professionals responsible for emotional care, with 65 (64.4%) of respondents supporting this view. Conversely, in the study by Kateroudaki et al. [8], nurses were more frequently cited as key providers of emotional support.

These discrepancies may stem from differences in healthcare settings, institutional protocols, or evolving perspectives on multidisciplinary collaboration in tracheostomy management. They underscore the importance of continually assessing and optimizing interdisciplinary roles to ensure comprehensive and effective patient care. Further research is needed to explore these variations and establish standardized guidelines for best practices in tracheostomy management.

Organizations must consider the implementation of multidisciplinary teams as a valuable investment that improves patient outcomes while offering significant long-term economic benefits. This requires overcoming challenges such as resource allocation, continuous training, and fostering collaboration and coordination among diverse professionals.

## Conclusions

This study aimed to explore healthcare professionals' perspectives on tracheostomy patient management. Understanding these perspectives can support the appropriate allocation of responsibilities and contribute to the development of a more structured, effective approach to care, guided by each professional’s training and clinical experience. The literature highlights a steady rise in tracheostomy cases, driven by various conditions requiring respiratory support. These cases demand complex, specialized care, involving physicians from different specialties and allied health professionals, underscoring the interdisciplinary nature of tracheostomy management. Despite their frequency and impact, there is a lack of standardized guidelines for addressing these patients’ diverse needs. This contributes to inconsistent practices and preventable complications, often leading to prolonged hospitalizations, ICU readmissions, increased healthcare costs, and elevated morbidity and mortality rates. The study emphasizes the need for coordinated, multidisciplinary care over siloed efforts.

Otolaryngologists (ENT specialists) are key in various procedures, such as determining indications, tube selection, managing complications, and patient education. However, other professionals also play critical roles: nurses manage direct care tasks like stoma care, oral hygiene, and suctioning; speech therapists aid communication; pulmonologists oversee respiratory function; dietitians advise on nutrition; and psychologists address emotional well-being. Findings highlight the importance of clinical experience and the current absence of clear role definitions in tracheostomy care. Healthcare institutions must take the lead in establishing multidisciplinary teams with specialized training and clear task distribution. By effectively managing resources and personnel, healthcare units promote comprehensive, patient-centered care, reduce complications, and enhance both patient outcomes and system sustainability. There is a clear need for further research on interdisciplinary care models, dedicated tracheostomy units, and the impact of team-based management on patient quality of life and healthcare economics. Moreover, the creation and adoption of international guidelines are crucial to ensure consistency and high standards of care across all healthcare settings.

## Appendices

**Table 2:** Questionnaire - Translated Version (English) The questionnaire was originally developed in Greek in 2015 by Chrysa Kateroudaki, Spyridoula Mourkogianni, and Mary Nikolaou as part of their undergraduate thesis, "The Views of Healthcare Professionals on Tracheostomy Management," at the Department of Speech Therapy, Faculty of Health and Welfare Professions, Technological Institute of Western Greece, in Patras (unpublished data). The questionnaire was provided with the written consent of its authors [8].

	QUESTIONNAIRE	
	A) Demographic Characteristics	
	Questions:	Answers:
1	Gender:	Male / Female
2	Age:	_ _ _ _
3	Level of education:	Τechnological Educational Insitute (TEI) / University (ΑΕΙ) / Master’s Degree / Doctorate
4	Profession/Specialization:	_ _ _ _
5	Years of professional practice:	_ _ _ _
6	Have you received special training regarding tracheostomy?	YES / NO
7	Do you stay updated on developments and the management of individuals with tracheostomy? If yes, how?	NO / YES - If yes, Source of information: Scientific articles / Internet / Books
8	When was the last time you read an article about tracheostomy?	A week ago / A month ago / A year ago / More than a year ago / I have not read a related article
9	Are you currently working or have you worked in the past with patients with tracheostomy?	YES / NO
10	How many patients with tracheostomy do you currently have?	1-2 patients / 3-4 patients / 5-6 patients / 7-8 patients / More than 8 / None
11	How would you describe your general knowledge regarding tracheostomy?	Excellent / Very good / Good / Average / Poor
	B) Healthcare professionals' opinions on who is best suited to handle 20 specific actions	
	Questions:	Answers:
1	Which professionals are capable of identifying the indications leading to tracheostomy placement?	Pulmonologist / Anesthesiologist / ENT Specialist / Surgeon / Physiotherapist / Occupational Therapist / Speech Therapist / Nurses / Dietitian / Other-Specify?
2	Which healthcare professionals are responsible for informing the patient about changes in respiratory physiology following the procedure?	Pulmonologist / Anesthesiologist / ENT Specialist / Surgeon / Physiotherapist / Occupational Therapist / Speech Therapist / Nurses / Dietitian / Other-Specify?
3	Who determines the internal/external diameter and length of the tracheostomy tube?	Pulmonologist / Anesthesiologist / ENT Specialist / Surgeon / Physiotherapist / Occupational Therapist / Speech Therapist / Nurses / Dietitian / Other-Specify?
4	Which professionals decide on the use of fenestrated tubes suitable for phonation?	Pulmonologist / Anesthesiologist / ENT Specialist / Surgeon / Physiotherapist / Occupational Therapist / Speech Therapist / Nurses / Dietitian / Other-Specify?
5	Which professionals decide on the type of cuff to be used?	Pulmonologist / Anesthesiologist / ENT Specialist / Surgeon / Physiotherapist / Occupational Therapist / Speech Therapist / Nurses / Dietitian / Other-Specify?
6	Which professionals handle the measurement (inflation or deflation) of cuff pressure?	Pulmonologist / Anesthesiologist / ENT Specialist / Surgeon / Physiotherapist / Occupational Therapist / Speech Therapist / Nurses / Dietitian / Other-Specify?
7	Who primarily works with the patient in the hospital ward?	Pulmonologist / Anesthesiologist / ENT Specialist / Surgeon / Physiotherapist / Occupational Therapist / Speech Therapist / Nurses / Dietitian / Other-Specify?
8	Who is responsible for managing the patient’s respiratory function through mechanical support?	Pulmonologist / Anesthesiologist / ENT Specialist / Surgeon / Physiotherapist / Occupational Therapist / Speech Therapist / Nurses / Dietitian / Other-Specify?
9	In case of complications with a tracheoesophageal fistula, who manages the situation?	Pulmonologist / Anesthesiologist / ENT Specialist / Surgeon / Physiotherapist / Occupational Therapist / Speech Therapist / Nurses / Dietitian / Other-Specify?
10	Who is responsible for tracheostomy site care?	Pulmonologist / Anesthesiologist / ENT Specialist / Surgeon / Physiotherapist / Occupational Therapist / Speech Therapist / Nurses / Dietitian / Other-Specify?
11	Who is called to perform suctioning for a patient with tracheostomy?	Pulmonologist / Anesthesiologist / ENT Specialist / Surgeon / Physiotherapist / Occupational Therapist / Speech Therapist / Nurses / Dietitian / Other-Specify?
12	Which healthcare professionals are responsible for educating and teaching hygiene and care rules for tracheostomy maintenance?	Pulmonologist / Anesthesiologist / ENT Specialist / Surgeon / Physiotherapist / Occupational Therapist / Speech Therapist / Nurses / Dietitian / Other-Specify?
13	Which specialists focus on oral hygiene for patients with tracheostomy?	Pulmonologist / Anesthesiologist / ENT Specialist / Surgeon / Physiotherapist / Occupational Therapist / Speech Therapist / Nurses / Dietitian / Other-Specify?
14	Which healthcare professionals are suitable to assess swallowing in a patient with tracheostomy?	Pulmonologist / Anesthesiologist / ENT Specialist / Surgeon / Physiotherapist / Occupational Therapist / Speech Therapist / Nurses / Dietitian / Other-Specify?
15	Which healthcare professionals are appropriate to explain cuff usage during feeding?	Pulmonologist / Anesthesiologist / ENT Specialist / Surgeon / Physiotherapist / Occupational Therapist / Speech Therapist / Nurses / Dietitian / Other-Specify?
16	Who deals with food consistency to ensure safe oral feeding for individuals with tracheostomy?	Pulmonologist / Anesthesiologist / ENT Specialist / Surgeon / Physiotherapist / Occupational Therapist / Speech Therapist / Nurses / Dietitian / Other-Specify?
17	Which healthcare professionals are responsible for ensuring the correct posture and head position during and after feeding?	Pulmonologist / Anesthesiologist / ENT Specialist / Surgeon / Physiotherapist / Occupational Therapist / Speech Therapist / Nurses / Dietitian / Other-Specify?
18	Who is responsible for caring for patients with communication problems due to tracheostomy?	Pulmonologist / Anesthesiologist / ENT Specialist / Surgeon / Physiotherapist / Occupational Therapist / Speech Therapist / Nurses / Dietitian / Other-Specify?
19	Which specialists are responsible for training the patient in alternative communication methods until normal function is restored?	Pulmonologist / Anesthesiologist / ENT Specialist / Surgeon / Physiotherapist / Occupational Therapist / Speech Therapist / Nurses / Dietitian / Other-Specify?
20	Which specialists provide emotional care to tracheostomy patients to address potential psychological issues?	Pulmonologist / Anesthesiologist / ENT Specialist / Surgeon / Physiotherapist / Occupational Therapist / Speech Therapist / Nurses / Dietitian / Other-Specify?

**Table 3:** Questionnaire - Original Version (Greek) The questionnaire was originally developed in Greek in 2015 by Chrysa Kateroudaki, Spyridoula Mourkogianni, and Mary Nikolaou as part of their undergraduate thesis, "The Views of Healthcare Professionals on Tracheostomy Management," at the Department of Speech Therapy, Faculty of Health and Welfare Professions, Technological Institute of Western Greece, in Patras (unpublished data). The questionnaire was provided with the written consent of its authors [8].

	ΕΡΩΤΗΜΑΤΟΛΟΓΙΟ	
	A) ΔΗΜΟΓΡΑΦΙΚΑ ΧΑΡΑΚΤΗΡΙΣΤΙΚΑ	
	Ερωτήσεις:	Απαντήσεις:
1	Φύλο:	Άντρας / Γυναίκα
2	Ηλικία:	_ _ _ _
3	Επίπεδο εκπαίδευσης:	ΤΕΙ / ΑΕΙ / Μεταπτυχιακό / Διδακτορικό
4	Επάγγελμα / Ειδικότητα:	_ _ _ _
5	Έτη που ασκείται το επάγγελμα σας:	_ _ _ _
6	Έχετε λάβει ειδική εκπαίδευση όσο αφορά την τραχειοστομία;	ΝΑΙ / ΌΧΙ
7	Ενημερώνεστε για τις εξελίξεις και την διαχείριση των ατόμων με τραχειοστομία; Αν ναι, με ποιο τρόπο;	ΌΧΙ / ΝΑΙ - Εάν ναι, Πηγή πληροφόρησης: Επιστημονικά άρθρα / Διαδίκτυο / Βιβλία
8	Πότε ήταν η τελευταία φορά που διαβάσατε ένα άρθρο σχετικά με την τραχειοστομία;	Πριν μια βδομάδα / Πριν ένα μήνα / Πριν ένα χρόνο / Περισσότερο από χρόνο / Δεν έχω διαβάσει σχετικό άρθρο
9	Δουλεύετε αυτή την περίοδο ή είχατε δουλέψει στο παρελθόν με ασθενείς με τραχειοστομία;	ΝΑΙ / ΌΧΙ
10	Πόσους ασθενείς έχετε σήμερα με τραχειοστομία;	1-2 ασθενείς / 3-4 ασθενείς / 5-6 ασθενείς / 7-8 ασθενείς / Πάνω από 8 / Κανέναν
11	Πως θα χαρακτηρίζατε τις γενικές γνώσεις σας όσο αφορά την τραχειοστομία;	Άριστες / Πολύ καλές / Καλές / Μέτριες / Καθόλου καλές
	B) Οι απόψεις των επαγγελματιών υγείας σχετικά με το ποιος είναι πιο κατάλληλος να διαχειριστεί 20 συγκεκριμένες ενέργειες.	
	Ερωτήσεις:	Απαντήσεις:
1	Ποιοι επαγγελματίες είναι σε θέση να ανιχνεύουν τις ενδείξεις που θα οδηγήσουν στη τοποθέτηση τραχειοστομίας;	Πνευμονολόγος / Αναισθησιολόγος / ΩΡΛ / Χειρουργός / Φυσικοθεραπευτής / Εργοθεραπευτής / Νοσηλευτής / Διαιτολόγος / Άλλος-Ποιος;
2	Ποιοι επαγγελματίες υγείας αναλαμβάνουν να ενημερώσουν τον ασθενή για τις αλλαγές που προκύπτουν στην φυσιολογία της αναπνευστικής λειτουργίας μετά την επέμβαση;	Πνευμονολόγος / Αναισθησιολόγος / ΩΡΛ / Χειρουργός / Φυσικοθεραπευτής / Εργοθεραπευτής / Νοσηλευτής / Διαιτολόγος / Άλλος-Ποιος;
3	Ποιος ασχολείται με την επιλογή της εσωτερικής/εξωτερικής διαμέτρου και μήκους του τραχειοσωλήνα;	Πνευμονολόγος / Αναισθησιολόγος / ΩΡΛ / Χειρουργός / Φυσικοθεραπευτής / Εργοθεραπευτής / Νοσηλευτής / Διαιτολόγος / Άλλος-Ποιος;
4	Ποιοι επαγγελματίες θα κρίνουν αν θα πρέπει να χρησιμοποιηθούν σωλήνες με παράθυρο κατάλληλο για παραγωγή φώνησης;	Πνευμονολόγος / Αναισθησιολόγος / ΩΡΛ / Χειρουργός / Φυσικοθεραπευτής / Εργοθεραπευτής / Νοσηλευτής / Διαιτολόγος / Άλλος-Ποιος;
5	Ποιοι επαγγελματίες θα κρίνουν την επιλογή του τύπου cuff;	Πνευμονολόγος / Αναισθησιολόγος / ΩΡΛ / Χειρουργός / Φυσικοθεραπευτής / Εργοθεραπευτής / Νοσηλευτής / Διαιτολόγος / Άλλος-Ποιος;
6	Ποιοι επαγγελματίες ασχολούνται με την μέτρηση (χορήγηση ή αφαίρεση αέρα) της πίεσης του cuff;	Πνευμονολόγος / Αναισθησιολόγος / ΩΡΛ / Χειρουργός / Φυσικοθεραπευτής / Εργοθεραπευτής / Νοσηλευτής / Διαιτολόγος / Άλλος-Ποιος;
7	Ποιοι από τους παρακάτω ασχολούνται περισσότερο με τον ασθενή στο θάλαμο νοσηλείας;	Πνευμονολόγος / Αναισθησιολόγος / ΩΡΛ / Χειρουργός / Φυσικοθεραπευτής / Εργοθεραπευτής / Νοσηλευτής / Διαιτολόγος / Άλλος-Ποιος;
8	Ποιος είναι υπεύθυνος για την διαχείριση της αναπνευστικής λειτουργίας του ασθενή μέσω μηχανικής υποστήριξης;	Πνευμονολόγος / Αναισθησιολόγος / ΩΡΛ / Χειρουργός / Φυσικοθεραπευτής / Εργοθεραπευτής / Νοσηλευτής / Διαιτολόγος / Άλλος-Ποιος;
9	Σε περίπτωση επιπλοκής με τραχειοοισοφαγικό συρίγγιο, ποιος διαχειρίζεται το περιστατικό;	Πνευμονολόγος / Αναισθησιολόγος / ΩΡΛ / Χειρουργός / Φυσικοθεραπευτής / Εργοθεραπευτής / Νοσηλευτής / Διαιτολόγος / Άλλος-Ποιος;
10	Ποιος αναλαμβάνει τη φροντίδα του στόματος της τραχείας;	Πνευμονολόγος / Αναισθησιολόγος / ΩΡΛ / Χειρουργός / Φυσικοθεραπευτής / Εργοθεραπευτής / Νοσηλευτής / Διαιτολόγος / Άλλος-Ποιος;
11	Ποιοι καλούνται να πραγματοποιήσουν την διαδικασία της αναρρόφησης σε έναν ασθενή με τραχειοστομία;	Πνευμονολόγος / Αναισθησιολόγος / ΩΡΛ / Χειρουργός / Φυσικοθεραπευτής / Εργοθεραπευτής / Νοσηλευτής / Διαιτολόγος / Άλλος-Ποιος;
12	Ποιοι επαγγελματίες υγείας είναι υπεύθυνοι για την ενημέρωση και διδασκαλία των κανόνων υγιεινής και φροντίδας που πρέπει να τηρούνται κατά την περιποίηση μιας τραχειοστομίας;	Πνευμονολόγος / Αναισθησιολόγος / ΩΡΛ / Χειρουργός / Φυσικοθεραπευτής / Εργοθεραπευτής / Νοσηλευτής / Διαιτολόγος / Άλλος-Ποιος;
13	Ποιοι ειδικοί ασχολούνται με την περιποίηση της στοματικής υγιεινής στους ασθενείς με τραχειοστομία;	Πνευμονολόγος / Αναισθησιολόγος / ΩΡΛ / Χειρουργός / Φυσικοθεραπευτής / Εργοθεραπευτής / Νοσηλευτής / Διαιτολόγος / Άλλος-Ποιος;
14	Ποιοι επαγγελματίες υγείας είναι κατάλληλοι ώστε να αξιολογήσουν την κατάποση ενός ασθενή με τραχειοστομία;	Πνευμονολόγος / Αναισθησιολόγος / ΩΡΛ / Χειρουργός / Φυσικοθεραπευτής / Εργοθεραπευτής / Νοσηλευτής / Διαιτολόγος / Άλλος-Ποιος;
15	Ποιοι επαγγελματίες υγείας είναι κατάλληλοι για να εξηγήσουν τη χρήση του cuff κατά τη διάρκεια της σίτισης στους ασθενείς;	Πνευμονολόγος / Αναισθησιολόγος / ΩΡΛ / Χειρουργός / Φυσικοθεραπευτής / Εργοθεραπευτής / Νοσηλευτής / Διαιτολόγος / Άλλος-Ποιος;
16	Ποιοι ασχολούνται με την σύσταση της τροφής όσο αφορά την ασφαλή σίτιση από το στόμα σε άτομα με τραχειοστομία;	Πνευμονολόγος / Αναισθησιολόγος / ΩΡΛ / Χειρουργός / Φυσικοθεραπευτής / Εργοθεραπευτής / Νοσηλευτής / Διαιτολόγος / Άλλος-Ποιος;
17	Ποιοι επαγγελματίες υγείας είναι υπεύθυνοι για την σωστή θέση και στάση του σώματος και της κεφαλής κατα την διάρκεια και μετά την σίτιση;	Πνευμονολόγος / Αναισθησιολόγος / ΩΡΛ / Χειρουργός / Φυσικοθεραπευτής / Εργοθεραπευτής / Νοσηλευτής / Διαιτολόγος / Άλλος-Ποιος;
18	Ποιος είναι υπεύθυνος για τη φροντίδα ασθενών που παρουσιάζουν προβλήματα επικοινωνίας λόγω της τραχειοστομίας;	Πνευμονολόγος / Αναισθησιολόγος / ΩΡΛ / Χειρουργός / Φυσικοθεραπευτής / Εργοθεραπευτής / Νοσηλευτής / Διαιτολόγος / Άλλος-Ποιος;
19	Ποιοι ειδικοί είναι υπεύθυνοι να εκπαιδεύσουν τον ασθενή με άλλα επικοινωνιακά μέσα μέχρις ότου η φυσιολογική λειτουργία να είναι εφικτή;	Πνευμονολόγος / Αναισθησιολόγος / ΩΡΛ / Χειρουργός / Φυσικοθεραπευτής / Εργοθεραπευτής / Νοσηλευτής / Διαιτολόγος / Άλλος-Ποιος;
20	Ποιοι ειδικοί παρέχουν συναισθηματική φροντίδα στους ασθενείς με τραχειοστομία για την εξομάλυνση τυχών ψυχολογικών καταστάσεων;	Πνευμονολόγος / Αναισθησιολόγος / ΩΡΛ / Χειρουργός / Φυσικοθεραπευτής / Εργοθεραπευτής / Νοσηλευτής / Διαιτολόγος / Άλλος-Ποιος;
